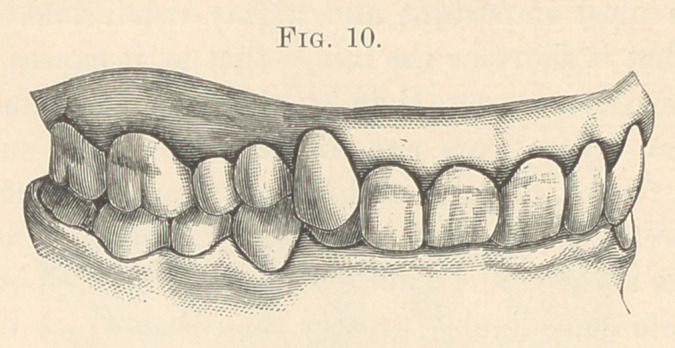# Consequences of the Extraction of Permanent Teeth

**Published:** 1900-05

**Authors:** E. A. Bogue

**Affiliations:** New York, N. Y.


### CONSEQUENCES OF THE EXTRACTION OF PERMANENT TEETH.[1]

BY E. A. BOGUE, M.D., NEW YORK, N. Y.

Mr. President, and Gentlemen of the Massachusetts State Dental Society,—A distinguished surgeon lately said to me that it seemed a pity that human life must be sacrificed to a considerable extent before any surgeon becomes thoroughly qualified to save life. A modification of this remark seems specially applicable in dentistry. It seems a pity that human teeth must be sacrificed, extensively, before the dental surgeon becomes thoroughly qualified to save them. A recent paper in the *Dental Cosmos,* from a gentleman who deprecates the formation of societies

---

[1] Read before the Massachusetts Dental Society, June 7 and 8, 1899.

[This paper has heretofore been published, but, with some changes and new illustrations, it is presented as part of the proceedings of the above Society.—Ed.]

of stomatology, saying that it seems like reaching out into regions that do not belong to the dental practitioner, advocates the extraction of the sixth-year molars. In the hope of showing the facts somewhat more clearly than the desultory observations of clinical practice usually show, permit me to draw your attention to a few of the unfortunate results of the extraction of permanent teeth.

Every gentleman present knows perfectly well that the first permanent molars erupt at somewhere near the sixth year of the child's life. It is this fact which has given these teeth the name of sixth-year molars. When fully erupted, these four teeth, two above and two below, sustain the jaws in their relative positions while the deciduous teeth are being shed and replaced by the permanent ones. What short-sighted policy to remove any of these four supports! And yet it has been done at so early a period that plates have been required for the growing child to masticate upon until the second permanent, or twelfth-year molars appear.

In order to comprehend more accurately what takes place when permanent teeth are extracted, we ought to have before us the skull of a child of six years with the temporary teeth in position and with the anterior plates of alveolus removed, that we may see the permanent teeth in process of development. We would see, in the first place, that the jaws at that age are not large enough to hold the permanent teeth in their proper arches. Hence they are packed, in regular irregularity, pending the growth of the jaws and the enlargement of their arches to such size as will admit of the permanent teeth, standing like the stones of an arch, each supporting the other by contact with that other, and all supported in their places by contact with the occluding surfaces of the antagonizing teeth of the other jaw. It should be borne in mind also that as the child grows, and the dental arches enlarge, they expand their alveoli in two ways,—from above down (and from below upward) and from before backward, antero-posteriorly. This is well proved by placing the impressions of the temporary teeth of any child upon the impressions of the permanent teeth of the same child after it shall have grown up. By looking carefully at the positions of the temporary teeth, we find an arch which is a pretty good horseshoe,—that is to say, fairly round from one end of the arch to the other,—and pretty nearly flat, or pretty nearly a straight line from before backward from the incisors to the farther end of the molars. This, by the way, is very much the arrangement that the artificial tooth-maker contributes to dental art when he makes a horseshoe plate, the grinding and cutting ends of whose teeth would all touch the table if laid upon it; and this is the arrangement produced generally by extracting the sixth-year molars. These temporary teeth, with the simple arch and straight lines, are sufficiently adapted to the mastication of the soft food that constitutes the child's nourishment, but they are not adapted to the necessities of adult life. If all the dental organs, temporary and permanent, are faithfully preserved to fulfil the functions of maturity, the twelfth-year molars of the lower jaw, developing upward, will be a little higher than the sixth-year molars, because they will have developed upon the curved lower jaw, which has grown backward and upward beyond what it was when it was the baby jaw. Following the line of that upward curve, the crown of the twelfth-year molar leans forward and pushes against the sixth-year molar at the point where the enamel is thickest, and this is at the greatest tuberosity of the tooth. The wisdom-tooth, when it appears regularly, rises still higher up the curve of the lower jaw than the twelfth-year molar, and leans against the twelfth-year molar in exactly the same relative position that the twelfth-year molar leans against the sixth; but, gentlemen, if that lower wisdom-tooth gets a faulty start and becomes impacted beneath the tuberosity of the twelfth-year molar, we, who have seen patients given up to die because of this trouble, do not need to be told that the developmental pressure from behind of an impacted wisdom-tooth and the forward push of the molars is very great indeed.

Now, to return to the upper jaw, the teeth in which regularly develop later than those below. As these upper teeth develop they descend, and their cusps are meshed in with the cusps of the lower teeth in a manner resembling the cogs of two wheels that intermesh. The upper teeth incline outward, the lower molars and bicuspids incline inward towards the tongue, and this results in the upper molars coming down astride of the outer row of cusps of the lower molars and bicuspids in such fashion that the largest portions of the superficial areas of these corrugated or cusped teeth are brought into contact and are enabled to slide against each other in the triturating movements of the jaw so as most effectually to comminute the food in the act of mastication.

Please to note carefully at this point that when the development of the molars is complete (and this comprises bicuspids or premolars as well), the curve of the lower jaw quite represents the concavity of a mortar, while the upper jaw may be imagined to be the convexity of the pestle, both reinforced by corrugations fitting into each other and adapted to do the heavy work of adult life. If now we imagine the removal of one stone or one brick from an arch, the imagination spontaneously sees the other members of this arch falling together. This is just what happens when one member of these complicated dental arches of sixteen members each is removed. Not only do the other members fall together in a different manner from the normal one, but that manner is in conformity to the pressure of development, which pushes from the wisdom-tooth forward to the cuspid, both above and below, while the force of the lips draws backward to the cuspids all incisor teeth above and below. The upper and lower cuspids, therefore, may be regarded as being *the most nearly fixed points* of any among the movable occupants of the mouth. In illustration of the operation of this fixed law of development, and that nature's efforts to repair

Fig. 1.

damage are always exerted along the same lines, I present for your consideration the drawings of two mouths, one of which has lost

Fig. 2.

the right lower bicuspid by extraction, the other of which lost the left lower bicuspid by failure to develop. The results are the same, and trituration cannot be performed upon the side where the ex-

FIG. 3.

FIG. 4.

traction or the loss occurred. Incidentally let me call attention to the fact that the size of the dental arches being diminished by the width of one member in each of these cases gives that much less room for the free movements of the tongue, and to the critical

FIG. 5.

FIG. 6.

observer that side of the face in each of these cases was noticeably smaller than the other. These same conditions appear in the drawings in the *Dental Cosmos* for June, 1899, pages 526 to 530, where all the models shown exhibit a straightening of the horizontal lines of mastication, a return towards the conditions of the temporary teeth, a consequent diminution in masticating power, a shortening of the bite, and, if we are permitted to allude to appearances, a weakening of the expression of the countenance.

A third case, which I present, also had the two first upper bicuspids removed and the upper front teeth drawn downward, spreading the cuspids somewhat. "This regulation took place when the patient was fourteen years of age, at which time, according to my description written then, the lower cuspid stood upright, the lower first bicuspid leaned a little backward, the second bicuspid a trifle forward, so that its tuberosity touched accurately the tuberosity of the first bicuspid. The first lower molar occupied a similar position towards the second bicuspid, and the second molar in turn the same thing towards the first. The meshing of the cusps between upper and lower teeth was good. Mastication and trituration could be performed on both sides of the mouth." The second model of this mouth was taken six years later. The lower incisors lean considerably forward, the lower cuspids are no longer upright, the first bicuspid leans so far forward that the tuberosity of the second bicuspid is below the tuberosity of the first, and almost the same condition exists between the molar and the second bicuspid. The lower arch is distinctly made narrower, and yet it will be remembered that nothing has been extracted from the lower jaw, nor has any tooth been filed. The movement that we see in this case is entirely due to the change in occlusion, which has taken place since removing the natural support from in front of the second bicuspid. We cannot see that the six upper front teeth have gone backward, although I tried my best to make them do so, but we do see that the back upper teeth have come forward, and in their coming forward the change in the lines of contact has been such that the upper teeth have driven the lower teeth forward also, and in that forward movement has occurred a distinct shortening of the bite to the extent seen here.

The third model of this mouth has been made within a few weeks, and is ten years later than the second model. A number of fractures have occurred of the teeth at various points owing to the malocclusion produced by those unfortunate extractions sixteen years ago. The next diagram shows a straightening of the horizontal lines of the dental arches from before backward, so that the concavity of the line of the lower molars and the convexity of the line of the upper molars is scarcely, if at all, perceptible, restoring very much the style of occlusion of the temporary teeth. These two cases have lost the twelfth-year molars.

FIG. 7.

FIG. 8.

Another result that follows the loss of permanent teeth is generally a diminishing of the arch of the six front teeth, and a straightening, because of the shortening of the lines of the back teeth. The reason for this is apparent when we consider that the individual members of an arch are seeking to support each other.

The third effect, distinctly visible if permanent teeth are early extracted, is a diminution in the size of the arch or vault of the palate, the effect of which is to interfere very seriously with vocalization by diminishing the boundary lines in which the vocal organs must necessarily act, so that supreme vocalization, whether by actor, clergyman, singer, or lawyer, is in its greatest perfection impossible where the destructive hand of the extractor has been at work. Patti would never have been heard of if her sixth-year molars had been extracted at an early age. The straightening of the lines of dental arches and the diminution of the size of the arch frequently leaves so little room for the tongue that accurate enunciation is rendered difficult if not impossible. This is one of the results that does not immediately supervene upon extraction, but comes later, and is therefore not as perceptible to the casual observer as some of the other results.

Again, a most important and highly deleterious result of extraction is that it shortens the bite,—that is, it causes the nose and chin to approximate more than would be normal had there been no extraction. Ten years ago, in discussing this subject with a professional friend, he presented for our inspection some models of the same mouth before and after the extraction of certain teeth, and asked me if the model after extraction did not show a lengthening of the bite considerably greater than that which existed before extraction. I was obliged to admit that there was lengthening, but the bite was not as long as it would normally have been had the extraction not been practised. One of our profession lately said, "The jaws are like scissors with something between. The least thing taken away allows the scissor-blades to come nearer." This is very concisely put, but an ocular demonstration of the truth of my assertion—that with the development of the permanent teeth occurs also an increased development of the alveoli of both jaws and a lengthening or opening of the bite consequent upon the eruption of the permanent teeth—is very plainly shown in the diagram

Fig. 9.

and several models which I present herewith, showing certain deciduous teeth caught at their original level and remaining after the development and consequent lengthening of the permanent teeth. A patient came into my hands many years ago whose bite was so shortened by the extraction of the sixth-year molars that the lower incisors were not at all visible when the mouth was closed, but were striking the upper gums just back of the incisors to such an extent that the mouth was constantly sore and giving pain. The molars and bicuspids were all wedged apart and approximal fillings that knuckled were put in, by this means preserving permanently, in a measure, the spaces that had been gained by wedging. This process lengthened the bite sufficiently to free

Fig. 10.

the gums from the contact of the lower incisors, and has remained an efficient remedy for twenty years. Occasionally after extraction of molar teeth the bite is so shortened that the lower incisors, striking behind and against the upper incisors, drive them forward and cause the upper incisors to project to such a degree that closure of the lips is difficult if not impossible. The only remedy for this condition of things is again to supply the missing teeth, or missing substance, thereby lengthening the bite and withdrawing the lower incisors from their contact with the upper ones. This is an operation that the more experienced men among us are sometimes called upon to perform.

Following upon this separation of certain teeth, where extraction has been practised, comes an undue crowding when the teeth touch at all. It is generally supposed, and, I think, is almost always said to the patient, that the extraction is done to make room. So far from making room, room is always diminished, and what room remains is crowded, not only by teeth touching too forcibly, but by their dropping inward in the straightened lines before spoken of towards the tongue, and impinging upon the room required by the tongue. If, a few years after extracting teeth, we were to take impressions of the same mouth, we should invariably find that room had been lost, not made.

Again, whenever the cutting or triturating ends of the teeth are not in contact, separation of certain teeth from each other is caused, leaving an exposure of the gums to the contact of hard food in the act of mastication. Another result incidental to these movements is the imperfect mastication of food owing to diminished surface and to the failure of the occluding surfaces of the upper and lower grinding teeth to mesh, thereby rendering thorough mastication of the food impossible. We are sometimes called a nation of dyspeptics, and the fault is ascribed to bad cookery. Perhaps it might be as well to ascribe some of it to bad dentistry, which, instead of faithfully preserving all of nature's powers, so often impairs or destroys the masticating power, and so sacrifices the best interests of the patient for the sake of a fee that ought to put to confusion him who receives it.

The next result that is distinctly noticeable is the wearing down of the cusps in those cases where extraction is practised. I have seen greater wear of the cusps at twenty or twenty-five years of age under those circumstances than ought to be found at fifty or sixty.

All the results that I have mentioned are plainly enough shown to those of us who will take the trouble to make and study plaster models of our patients' mouths.

A further result of extraction is the greater liability to the deposit of tartar at points where the self-cleansing conditions no longer exist. This arises sometimes from the lower incisor teeth being caused to incline inward instead of outward, as they ought, in which case it is made difficult, if not impossible, to effectually guard against the deposit of tartar or of food. There is also greater liability to a profound deposit of tartar, even to the formation of calcic abscess, on the sides of teeth that are not quite in contact. All interference with the arches, whether by extracting or filing teeth, favors the deposit of tartar, not only by forming a nidus of tranquillity of the oral fluids, but by preventing that trituration, which by itself very often tends to cleanse the teeth from deposits of food or of tartar.

Another result of extraction is the withdrawal of the normal support of the teeth constituting the arch, with the consequent destruction of a portion of the support of the normal socket.

The final result that I will notice on this occasion is the rotation on their axes of teeth posterior to the one extracted, which always gives a bad occluding surface to the antagonizing tooth in the opposite jaw and a triangular space more or less pronounced for the deposit of food and tartar.

During the last sixteen years or thereabouts, so far as I glean from my records, I have only caused four firm teeth to be extracted. Three of these were impacted wisdom-teeth; the fourth was a lateral incisor standing so far out of line as to mar an otherwise pleasant countenance. This incisor had resisted the efforts of several of our profession to coax it into place, and I considered myself justified in removing it, drawing the other incisors down to fill the vacancy.

I am sure that if my professional brethren will study the results of extraction, as I have been compelled to study them, from the failures that have resulted through extraction, their practices can scarcely be made as conservative as my own has been, and they will never extract unless the good to be gained will surely and greatly overbalance the injury that is sure to be done.

As an instance of what can be done in the way of regulating without extraction, I present diagrams of the teeth of a patient of my friend Dr. Baker, both before and after regulating, together with photographs of the patient before the operation, and several years after. The operations for regulating were all completed in a few weeks and without extracting any teeth, all of which is clearly shown by the illustrations. The success of this effort at regulating has, I think, converted Dr. Baker to the doctrine of regulating without extraction; as a rule, that has very few if any exceptions.

## Figures and Tables

**Fig. 1. f1:**
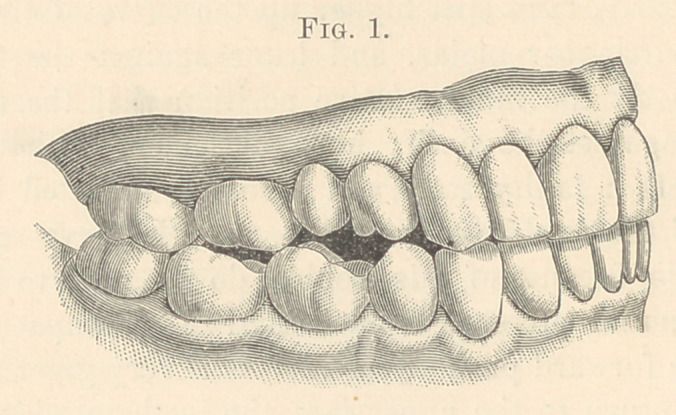


**Fig. 2. f2:**
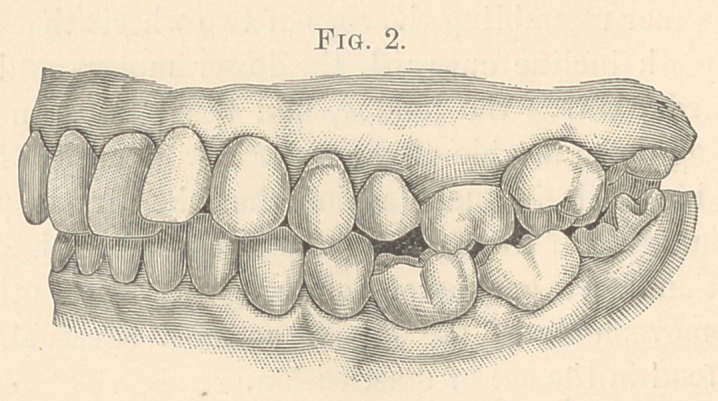


**Fig. 3. f3:**
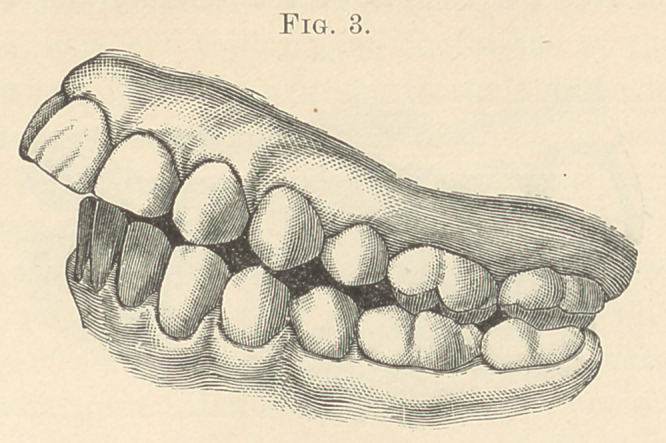


**Fig. 4. f4:**
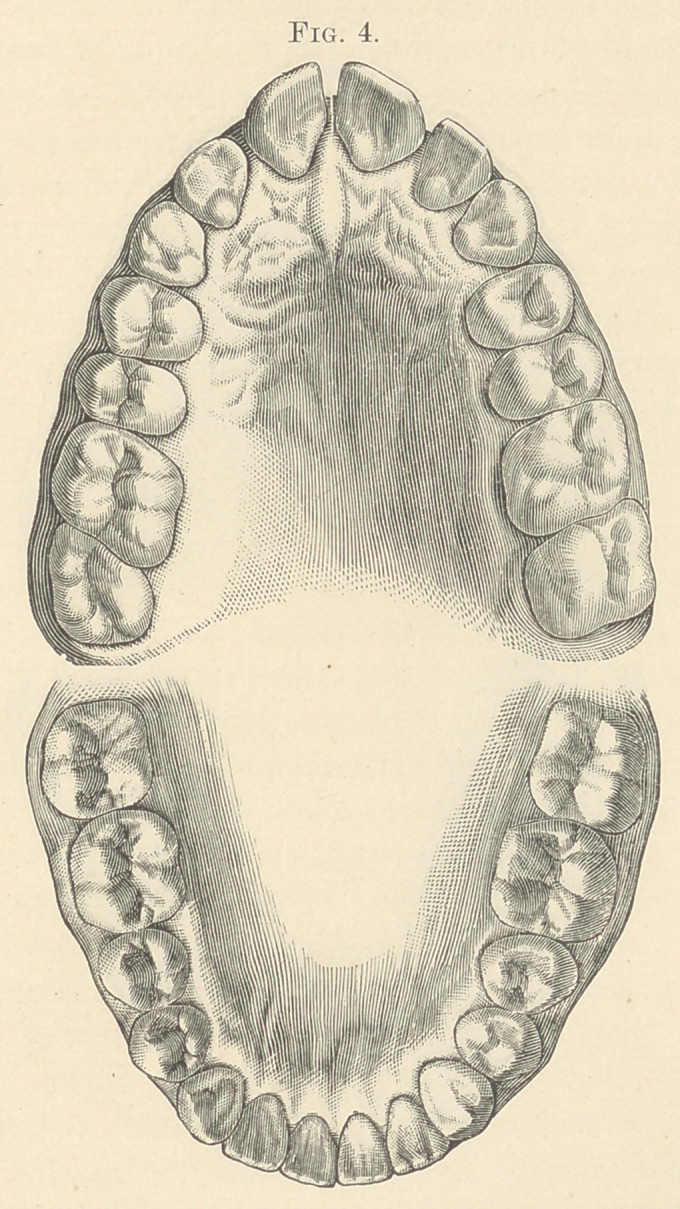


**Fig. 5. f5:**
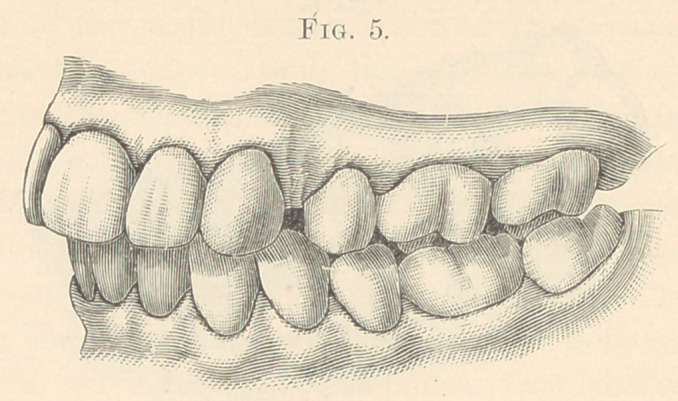


**Fig. 6. f6:**
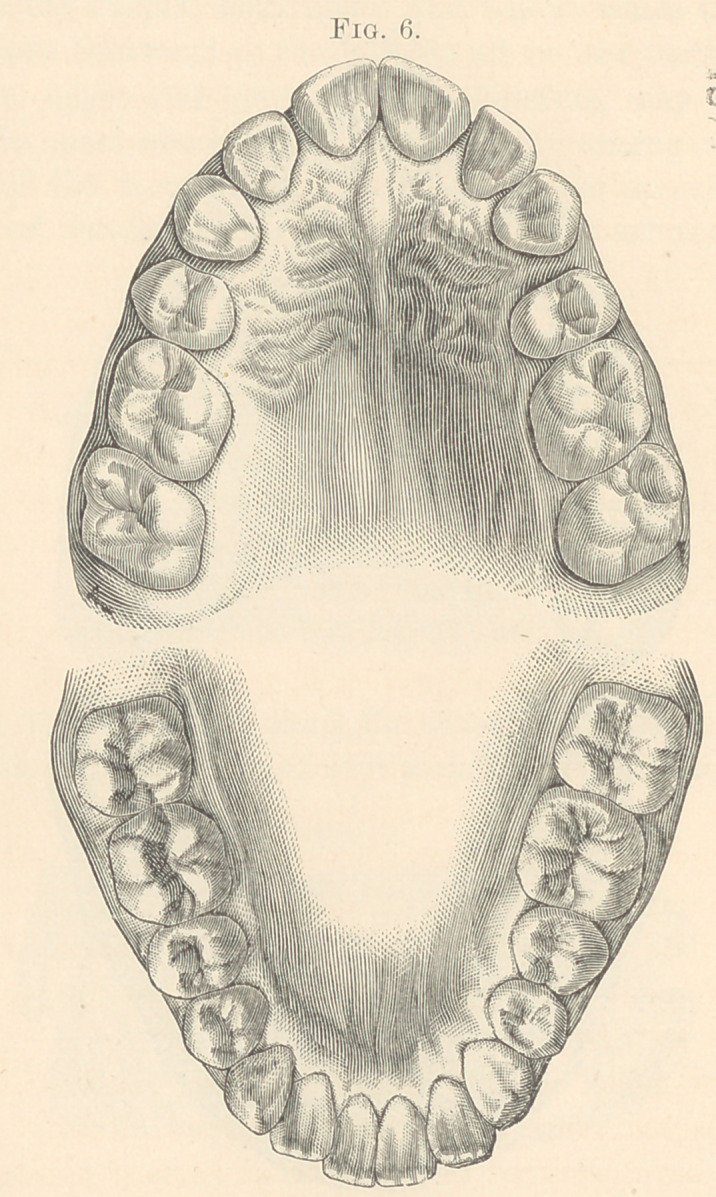


**Fig. 7. f7:**
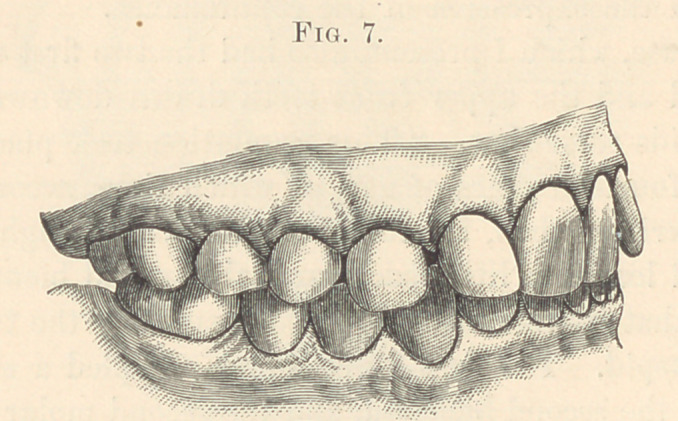


**Fig. 8. f8:**
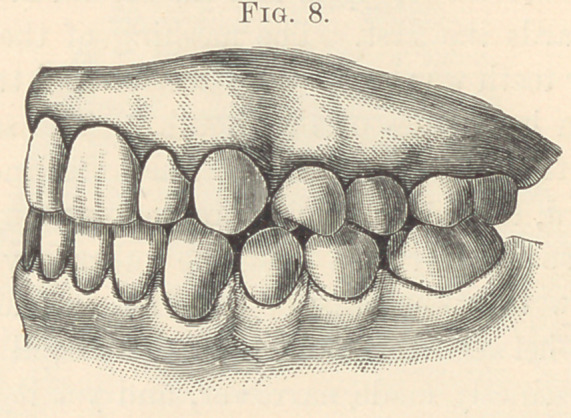


**Fig. 9. f9:**
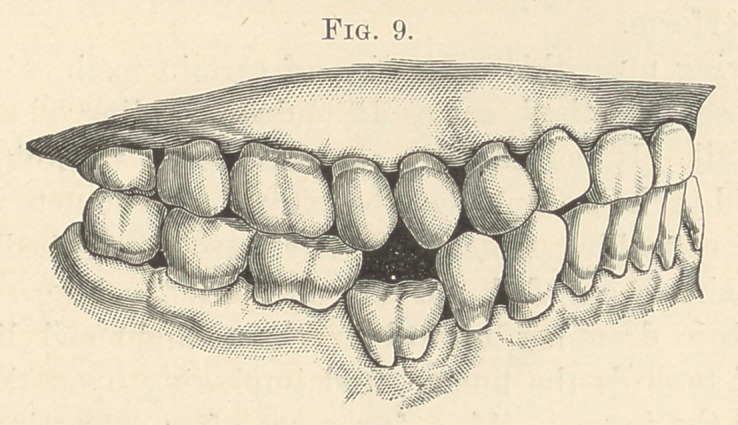


**Fig. 10. f10:**